# The fungal pattern recognition receptor, Dectin-1, and the associated cluster of C-type lectin-like receptors

**DOI:** 10.1111/j.1574-6968.2008.01418.x

**Published:** 2008-11-20

**Authors:** Cristal Huysamen, Gordon D Brown, Derek Sullivan

**Affiliations:** Clinical Laboratory Sciences, Division of Immunology, Institute of Infectious Disease and Molecular Medicine, University of Cape TownCape Town, South Africa

**Keywords:** C-type lectin, myeloid cells, ITAM, ITIM, signaling, homeostasis

## Abstract

The mammalian natural killer gene complex (NKC) contains several families of type II transmembrane C-type lectin-like receptors (CLRs) that are best known for their involvement in the detection of virally infected or transformed cells, through the recognition of endogenous (or self) proteinacious ligands. However, certain CLR families within the NKC, particularly those expressed by myeloid cells, recognize structurally diverse ligands and perform a variety of other immune and homoeostatic functions. One such family is the ‘Dectin-1 cluster’ of CLRs, which includes MICL, CLEC-2, CLEC12B, CLEC9A, CLEC-1, Dectin-1 and LOX-1. Here, we review each of these CLRs, exploring our current understanding of their ligands and functions and highlighting where they have provided new insights into the underlying mechanisms of immunity and homeostasis.

## Introduction

C-type lectins are a superfamily of proteins that contain one or more C-type lectin-like domains (CTLDs), and have been divided into 17 groups based on their phylogeny and domain organization (Zelensky & Gready, 2005). The CTLD consists of a distinctive protein fold that is generated through disulfide linkages between conserved cysteine residues, but despite the similarity in structure, these domains recognize diverse and structurally unrelated ligands. These receptors can be loosely classified as ‘classical’ and ‘nonclassical’ C-type lectins based on their ability to recognize carbohydrate and noncarbohydrate ligands, respectively. In addition, these receptors can be either membrane bound or soluble, being secreted from cells into the extracellular milieu.

**Fig. 1:**
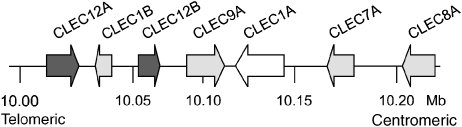
Cartoon representation of the genomic organization of the ‘Dectin-1 cluster’ in the NKC on human chromosome 12. Activation receptors are shown in light grey, inhibitory receptors in dark grey, and those whose function is unclear are shown in white.

**Fig. 2:**
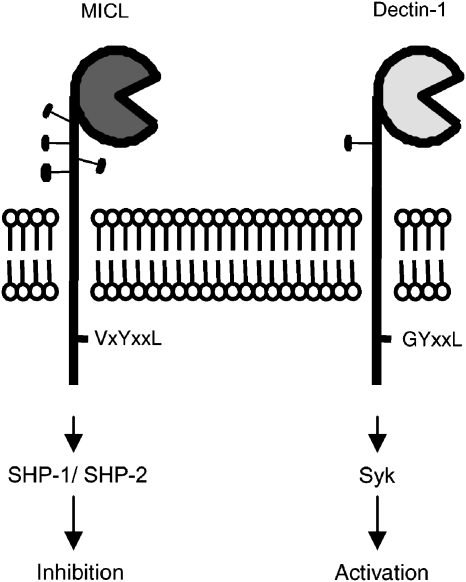
Cartoon representation of the typical structure of group V CLRs. Dectin-1 and MICL are shown as representatives of activation and inhibitory receptors that are found in this cluster. Proximal signaling components are also indicated. Lollipop structures indicate sites of N-linked glycosylation.

Of particular interest are the group V transmembrane C-type lectins encoded within the natural killer gene complex (NKC), on human chromosome 12 and mouse chromosome 6, which are best known for their recognition of endogenous proteinacious ligands and involvement in the detection of virally infected or transformed cells (Yokoyama & Plougastel, 2003). However, some CLR subfamilies, especially those expressed by myeloid cells, recognize structurally diverse ligands and perform a variety of other immune and homoeostatic functions, and are often encoded within a single gene cluster. One such subfamily is the ‘Dectin-1 cluster’ which is primarily, but not exclusively, expressed by myeloid cells [macrophages, dendritic cells (DC) and neutrophils] (Fig. 1). These type II transmembrane receptors, which include MICL, CLEC-2, CLEC12B, CLEC9A, CLEC-1, Dectin-1 and LOX-1, comprise a single extracellular CTLD, a stalk region of variable length and a cytoplasmic tail containing various signaling motifs (Fig. 2). Here, we will briefly review each of these CLRs in the order they are found in the genome (Fig. 1), highlighting their roles in immunity and homeostasis. A summary of the functions, ligands and expression of each receptor is shown in Table 1.

**Table 1:** Selected ligands and expression profiles of the Dectin-1 cluster of C-type lectin receptors

Official name	Alternate names	Cellular expression (human)	Exogenous ligands	Endogenous ligands	Cellular function
CLEC12A	MICL, DCAL2, CLL1, KLRL1	Myeloid cells	?	Yes but identity unknown	Inhibition
CLEC1B	CLEC-2	Platelets Myeloid cells?	Rhodocytin, HIV	Podoplanin	Activation
CLEC12B	Macrophage antigen H	Macrophages	?	?	Inhibition
CLEC9A	DNGR1	BDCA3^+^ DC, monocyte subsets, B-cells	?	?	Activation
CLEC1A	CLEC-1	DC	?	?	?
CLEC7A	Dectin-1	Myeloid cells, T-cell subsets, B cells, mast cells, eosinophils	β-Glucan, Mycobacterial ligand	T-cells Apoptotic cells	Activation
CLEC8A	LOX-1	Endothelium, smooth muscle, platelets, fibroblasts, macrophages	Gram-positive Gram-negative bacteria	ox-LDL modified lipoprotiens Aged/apoptotic cells Advanced glycation end-products HSP70	Activation

‘?’ refers to unknown data.

## Myeloid inhibitory C-type lectin-like receptor (MICL) (CLEC12A)

MICL was originally identified by homology to Dectin-1 and its position within the Dectin-1 cluster (Marshall *et al.*, 2004). MICL was subsequently identified by three other groups and named C-type lectin-like molecule-1 (CLL-1), dendritic-cell-associated C-type lectin 2 (DCAL-2) and killer cell lectin-like receptor 1 (KLRL1) (Bakker *et al.*, 2004; Han *et al.*, 2004; Chen *et al.*, 2006). Human MICL is variably spliced, generating at least three isoforms, and highly *N*-glycosylated, and although it contains cysteine residues in its stalk region, the receptor appears to be expressed as a monomer. Murine MICL, however, is expressed as a dimer and is not heavily glycosylated (Pyz *et al.*, 2008). Both murine and human MICL are predominantly expressed by myeloid cells (granulocytes, monocytes, macrophages and DCs) and expression of this receptor is downregulated following inflammatory conditions, including those triggered by microbial components (Marshall *et al.*, 2004, 2006; Pyz *et al.*, 2008). In humans, MICL has been identified as a marker for acute myeloid leukemia (Bakker *et al.*, 2004).

MICL contains a single ITIM in its cytoplasmic tail that can associate with the signaling phosphatases SHP-1 and SHP-2 (Han *et al.*, 2004; Marshall *et al.*, 2004; Pyz *et al.*, 2008), and is able to inhibit cellular activation (Marshall *et al.*, 2004). MICL has also been reported to inhibit NK cell cytotoxicity (Han *et al.*, 2004), although the receptor is not expressed on NK cells (Marshall *et al.*, 2006), and antibody-mediated cross-linking of MICL on human monocyte-derived DCs has been shown to affect DC function, by altering cytokine production, cellular maturation and chemokine receptor expression, suggesting a role for this receptor in the control of immune responses (Chen *et al.*, 2006). However, the mechanisms and significance of these effects are unclear. Recently murine MICL was shown to recognize an endogenous ligand that was present in many tissues, suggesting that the receptor may also play a role in homeostasis, but the identity of this molecule has yet to be determined (Pyz *et al.*, 2008).

## CLEC-2 (CLEC1B)

CLEC-2 was identified from a computational screen for CLRs at the same time as CLEC-1 (discussed below) (Colonna *et al.*, 2000). The expression of CLEC-2 has been described on platelets, megakaryocytes and liver sinusoidal endothelial cells, although reverse transcriptase (RT)-PCR analysis indicates that the receptor may also be expressed on monocytes, DC and granulocytes (Colonna *et al.*, 2000; Chaipan *et al.*, 2006; Suzuki-Inoue *et al.*, 2006). CLEC-2 is differentially glycosylated and, in mouse, is expressed in at least three isoforms, which are generated by alternative splicing and which possess different expression profiles and subcellular localizations (Suzuki-Inoue *et al.*, 2006; Xie *et al.*, 2008). Furthermore, full-length mCLEC-2 is proteolytically cleaved from the membrane, generating a soluble homodimeric form (Xie *et al.*, 2008).

Activation of CLEC-2 triggers platelet activation and aggregation, an activity that is mediated by the cytoplasmic immunoreceptor tyrosine-based activation (ITAM)-like motif of CLEC-2 (Suzuki-Inoue *et al.*, 2006). This single tyrosine-based motif is highly similar to that found in Dectin-1, and can recruit and signal via spleen tyrosine kinase (Syk) (Suzuki-Inoue *et al.*, 2006). In addition to Syk, Tec family kinases, Src, PLCγ and Rac1 have been implicated in the signaling pathway activated by CLEC-2 (Fuller *et al.*, 2007; Pleines *et al.*, 2008).

A number of endogenous and exogenous ligands for CLEC-2 have been identified, including the snake venom toxin, rhodocytin (Suzuki-Inoue *et al.*, 2006). Rhodocytin assembles as a tetramer and it is thought that recognition by CLEC-2 induces clustering of the receptor, triggering downstream signaling and platelet activation (Watson *et al.*, 2007, 2008; Hooley *et al.*, 2008). CLEC-2 can also bind HIV-1, and may capture and transfer infectious HIV-1 in cooperation with DC-SIGN (dendritic cell-specific intercellular adhesion molecule-3-grabbing non-integrin) (Chaipan *et al.*, 2006).

One endogenous ligand, podoplanin, has also been identified for CLEC-2 (Suzuki-Inoue *et al.*, 2007). Podoplanin is a mucin-type sialoglycoprotein that is expressed on a variety of cell types, but not on blood vessel endothelium, and has been implicated in tumor cell-induced platelet aggregation, tumor metastasis and lymphatic vessel formation. It has been suggested that the interaction of CLEC-2 with podoplanin, which involves *O*-glycans, may be involved in promoting tumor growth and/or metastasis, and could therefore be a target for therapeutic intervention (Suzuki-Inoue *et al.*, 2007; Christou *et al.*, 2008; Kato *et al.*, 2008). Despite the identification of all these ligands, however, the physiological role of CLEC-2 is still unknown.

## CLEC12B

Little is known about CLEC12B, which was identified in the Dectin-1 cluster through computational analysis (Sobanov *et al.*, 2001). RT-PCR analysis has indicated that human CLEC12B is widely expressed at low levels in various human tissues, except the brain, and that the receptor is alternatively spliced, generating at least two isoforms, one of which lacks part of the carbohydrate recognition domain (CRD) and would be predicted to be nonfunctional (Hoffmann *et al.*, 2007). This nonfunctional isoform appears to be preferentially expressed in certain tissues, including the lung, mammary gland and ovary. Although the receptor was not detected on any peripheral blood leukocyte, its expression was induced upon the differentiation of monocytes into macrophages (Hoffmann *et al.*, 2007). CLEC12B possesses an ITIM sequence (VxYxxL) within its cytoplasmic tail, which can recruit the inhibitory phosphatases, SHP-1 and SHP-2, and, using a model system, was shown to be able to inhibit certain cellular functions. More analysis of CLEC12B is required to understand its physiological role.

## CLEC9A

CLEC9A (which has also been termed DNGR1) is the most recently characterized receptor from the Dectin-1 cluster and is expressed in many tissues, as determined by RT-PCR analysis (Caminschi *et al.*, 2008; Huysamen *et al.*, 2008; Sancho *et al.*, 2008). In peripheral blood, human CLEC9A is found primarily on the surface of BDCA3^+^ DCs, although this receptor was also detected on small subsets of monocytes and B-cells. There are at least five isoforms of CLEC9A in mice, generated through alternative splicing, and the receptor is expressed on CD8^+^ DCs, which are thought to be the equivalent of human BDCA3^+^ cells, and also at low levels on plasmacytoid DC. Human CLEC9A and selected murine isoforms are expressed at the cell surface as glycosylated dimers.

The cytoplasmic tail of CLEC9A contains a single tyrosine residue within a sequence showing similarity to the ITAM-like sequence of Dectin-1, suggesting that CLEC9A may function as an activation receptor. Indeed, using receptor chimeras in transfected myeloid cells, CLEC9A was shown to be capable of inducing inflammatory cytokine production and signal via Syk kinase (Huysamen *et al.*, 2008). Although this receptor did not mediate particle uptake via phagocytosis, CLEC9A was capable of internalizing bound antigens via endocytosis and directing them to the endosomal/lysosomal pathway (Huysamen *et al.*, 2008; Sancho *et al.*, 2008). Interestingly, antigens targeted to CLEC9A could induce humoral, CD4^+^ and CD8^+^ T-cell responses, even in the absence of adjuvant, suggesting that this receptor could be exploited as a novel target on CD8^+^ or BDCA3^+^ DCs to drive these responses (Caminschi *et al.*, 2008; Sancho *et al.*, 2008). In fact, in a murine model, the cross-presentation of tumor antigens targeted to CLEC9A was found to elicit potent antitumor responses (Sancho *et al.*, 2008). However, the ligand(s) and physiological function of this receptor are still unknown.

## CLEC-1 (CLEC1A)

Virtually nothing is known about CLEC-1, a receptor originally identified through a computational search for NK-related CLRs (Colonna *et al.*, 2000). From analysis of tissue mRNA, the human receptor was shown to be expressed in the placenta, lung, bone marrow, thymus and heart. At a cellular level, the receptor was detected by RT-PCR in unstimulated as well as tumor necrosis factor (TNF) or CD40L-stimulated DCs, and in endothelial cells. However, CLEC-1 was not detected in peripheral blood monocytes (PBMCs), granulocytes, B, T, NK cells or monocytes (Colonna *et al.*, 2000; Sobanov *et al.*, 2001). CLEC-1 may be expressed in at least two isoforms, and the receptor contains a cytoplasmic tyrosine through which it may induce intracellular signaling, although this residue is not within a recognizable signaling motif. Unlike other members of the Dectin-1 cluster, however, CLEC-1 is not expressed at the cell surface, at least in transfected cells.

## Dectin-1 (CLEC7A)

The β-glucan receptor, Dectin-1, is one of the best characterized receptors in the ‘Dectin-1 cluster’ and was originally identified as a DC-specific molecule, from which its name ‘dendritic-cell-associated C-type lectin-1’ was derived (Ariizumi *et al.*, 2000; Brown, 2006). Dectin-1 is alternatively spliced generating two major and a number of minor isoforms, which have different functionalities, and the receptor is *N*-glycosylated, a posttranslation modification that contributes to its surface expression and function. Dectin-1 is predominantly expressed on monocytes, macrophages, neutrophils and microglia, but also weakly on a subset of T cells, and in humans, B cells, mast cells and eosinophils (Brown, 2006).

Dectin-1 specifically recognizes (1,3)-linked β-glucans in a calcium-independent manner and is the primary receptor for these carbohydrates on leukocytes (Brown, 2006; Palma *et al.*, 2006; Adams *et al.*, 2008). The crystal structure of the CTLD of Dectin-1 has been determined and mutational analyses have indicated that two residues (Trp^221^ and His^223^) are essentially required for β-glucan binding, and these two residues flank a groove on the CTLD that could be the binding site (Adachi *et al.*, 2004; Brown *et al.*, 2007). However, the exact mechanism of β-glucan recognition by Dectin-1 is still unknown.

Recognition of β-glucans by Dectin-1 leads to the induction of numerous cellular responses, including the respiratory burst, ligand uptake by endocytosis and phagocytosis, the production of arachidonic acid metabolites, DC maturation and the induction of numerous cytokines and chemokines, including TNF, CXCL2, IL-23, IL-6, IL-10 and IL-2 (Brown, 2006). Signaling from Dectin-1 is sufficient for many of these responses, but others, such as the respiratory burst and proinflammatory cytokine production, require, or are enhanced by, cooperative signaling from Myd88-coupled TLRs (Brown, 2006; Dennehy *et al.*, 2008). However, the ability of Dectin-1 to induce DC maturation and the production of cytokines, such as IL-23, can directly couple innate and adaptive immunity, independently of the TLRs (Leibundgut-Landmann *et al.*, 2007). Recent data also suggest that collaborative signaling between Dectin-1 and MyD88-coupled TLRs is far more extensive than first appreciated, and results in the enhanced production of IL-23, while downregulating the production of IL-12 (Gerosa *et al.*, 2008).

The ability of β-glucans to induce these cellular responses is also cell-type dependent. In macrophages (Dennehy *et al.*, 2008), for example, particulate β-glucans are unable to stimulate TNF production without costimulation of the TLRs, yet in DCs, β-glucan alone is sufficient to trigger the production of this cytokine (Leibundgut-Landmann *et al.*, 2007). Although the underlying reasons for these differences are not yet fully understood, they have recently been shown to be related, in part, to phagocytosis and the actions of cytokines, such as granulocyte monocyte colony stimulating factor (GM-CSF) (Rosas *et al.*, 2008).

Signaling from Dectin-1 is mediated by the cytoplasmic tail, which contains an ITAM-like motif. Upon ligand binding, this motif becomes tyrosine phosphorylated by Src family kinases, leading to the recruitment of Syk, which initiates subsequent downstream signaling pathways. The interaction between Syk and Dectin-1 is unusual, in that it involves only one tyrosine, and is not fully understood, but may involve the bridging of two Dectin-1 monomers (Rogers *et al.*, 2005). This type of interaction is now known to occur in at least two other Dectin-1 cluster receptors, CLEC-2 and CLEC9A. Downstream signaling involves the novel adaptor CARD9, as well as activation of MAP kinases, NFAT and nuclear factor-kappa B (NFκB) (Rogers *et al.*, 2005; Gross *et al.*, 2006; Goodridge *et al.*, 2007; Slack *et al.*, 2007) There are also Syk-independent pathways of Dectin-1 signaling, which may be cell-type specific, but the signaling mechanisms involved in these responses are largely uncharacterized (Brown, 2006).

Dectin-1 has been shown to recognize several fungal species, including *Saccharomyces, Candida, Pneumocystis, Coccidiodes, Penicillium* and *Aspergillus*, but not *Cryptococcus* (Brown, 2006; Nakamura *et al.*, 2007, 2008). Recognition of these organisms by Dectin-1 triggers many protective responses, such as fungal uptake by phagocytosis and killing via the respiratory burst. These interactions also induce the production of cytokines and chemokines, and while many of these are known to be protective in fungal infections (such as TNF, CXCL2, IL-1β, IL-1α, CCL3, GM-CSF, G-CSF and IL-6), others (such as IL-10 and IL-23) are paradoxically nonprotective (Brown, 2006). Although the reason for production of these nonprotective cytokines is not yet clear, there may be immunological benefits. IL-10, for example, inhibits antifungal immunity yet may be required to limit inflammatory pathology and to promote fungal persistence and long-term immunity.

The role of Dectin-1 has been examined *in vivo*, and although the data are not entirely consistent, they support a role for this receptor in antifungal immunity. Dectin-1-deficient mice, on a 129Sv background, displayed increased susceptibility to systemic infection with *Candida albicans*, which resulted from inflammatory defects and reduced fungal killing (Taylor *et al.*, 2007). However, Dectin-1 knockout mice on a C57BL6 background were not susceptible to *Candida*, but were found to show differences in fungal burdens during infection with *P. carinii*, which resulted from defects in the respiratory burst (Saijo *et al.*, 2007). However, C57BL6 mice deficient in CARD9, a downstream signaling component of Dectin-1, were susceptible to candidiasis, although this may also involve signaling pathways induced from other receptors (Gross *et al.*, 2006). Furthermore, in wild-type C57BL6 mice, inhibition of Dectin-1 during pulmonary challenge with *Aspergillus fumigatus* resulted in reduced lung inflammation and increased fungal burden (Steele *et al.*, 2005). In line with these findings, differential expression of Dectin-1 isoforms in the various mouse strains has also been linked to susceptibility to *Coccidoides* (Del Pilar Jimenez *et al.*, 2008).

Further support for a role in Dectin-1 in antifungal immunity comes from the growing body of evidence which indicates that fungal pathogens may actively avoid immune recognition by masking their β-glucans (Gantner *et al.*, 2005). Hyphae of *Aspergillus* and *Candida*, for example, do not expose β-glucans on their surface and enhancing the exposure of these carbohydrates, through treatment with caspofungin, has been shown to improve antifungal responses (Wheeler & Fink, 2006). Other examples include Histoplasma, which masks its β-glucan under a layer of α-glucan, and Paracoccidioides, which switches from β-glucan to α-glucan upon infection of the host.

Dectin-1 may also play other roles in addition to antifungal immunity. Three recent publications have suggested that Dectin-1 can recognize an unidentified ligand on mycobacteria, promoting bacterial uptake and the induction of a number of cytokines and chemokines, including TNF, IL-6, RANTES and G-CSF (Yadav & Schorey, 2006; Rothfuchs *et al.*, 2007; Shin *et al.*, 2008). These interactions also induce the production of IL-12 (Rothfuchs *et al.*, 2007), which is of particular interest, as this T-helper type 1-promoting cytokine is essential for the control of mycobacterial infection.

Dectin-1 can also recognize an endogenous ligand on T-cells, stimulating cellular activation and proliferation, and may therefore act as a costimulatory molecule (Brown, 2006). This function of Dectin-1 is supported by its expression on antigen presenting cells in the T-cell areas of lymphoid tissues. The recognition of the endogenous ligand has also recently been shown to mediate the recognition and uptake of apoptotic cells, and the cross-presentation of cellular antigens (Weck *et al.*, 2008). Recognition of the endogenous ligand occurs at a distinct binding site on Dectin-1, as its binding is not inhibitable by β-glucans. However, the nature of the endogenous ligand, which may be a protein, is still unknown.

## Lectin-like oxidized LDL receptor (LOX-1) (CLEC8A)

LOX-1 was the first member of the Dectin-1 cluster to be identified and is well characterized, being originally isolated from a bovine aortic endothelial cDNA expression library screened for receptors for oxidized LDL (OxLDL) (Sawamura *et al.*, 1997). LOX-1 is glycosylated, a posttranslational modification that contributes to cell-surface expression and ligand recognition, and the receptor forms homodimers that may multimerize through noncovalent interactions involving the neck region, aiding in ligand binding (Mehta *et al.*, 2006; Dunn *et al.*, 2008). LOX-1 can also be prototypically cleaved at the membrane proximal sites in the neck domain, producing a soluble form whose function is unknown. LOX-1 is expressed on vascular endothelial cells, smooth muscle cells, platelets, fibroblasts and macrophages and its expression can be upregulated by a variety of proinflammatory, oxidative and mechanical stimuli and during several pathological conditions *in vivo*, such as diabetes, hyperlipidemia, atherosclerosis and hypertension (reviewed in Chen & Du, 2007; Dunn *et al.*, 2008). Importantly, expression of LOX-1 can also be upregulated following binding of OxLDL, which may exacerbate the development of LOX-1-mediated diseases, such as atherosclerosis.

Although a part of the Dectin-1 C-type lectin cluster, LOX-1 is considered to be a member (class E) of the scavenger receptor family. In addition to OxLDL, LOX-1 recognizes numerous other structurally diverse ligands including modified lipoproteins, selected anionic polymers and phosphopolipids, aged and apoptotic cells, activated platelets, advanced glycation endproducts, heat shock protein (HSP)70, and gram-positive and gram-negative bacteria (Chen & Du, 2007). Ligand recognition is thought to involve electrostatic interactions with positively charged residues that are exposed on the face of the CRD of LOX-1 with negatively charged regions in the ligands (Mehta *et al.*, 2006; Dunn *et al.*, 2008).

Despite lacking classical signaling motifs in its cytoplasmic tail, LOX-1 can mediate or modulate a variety of cellular functions, including endocytosis, phagocytosis, cytokine production, CD40 and CD40 ligand levels, apoptosis, the activation of NFκB and production of reactive oxygen species (Mehta *et al.*, 2006; Chen *et al.*, 2007; Dunn *et al.*, 2008). LOX-1 can also act as a cell-adhesion molecule involved in leukocyte recruitment during inflammation and, through its ability to recognize HSP70, has been implicated in DC-mediated antigen cross-presentation (Delneste *et al.*, 2002; Mehta *et al.*, 2006; Dunn *et al.*, 2008). Although the signaling pathways leading to these responses are not fully understood, various downstream components have been implicated, including phosphoinositide 3-kinase, p38 mitogen-activated protein kinase and protein kinase Cα. Recently, LOX-1-mediated internalization of Ox-LDL was shown to occur through a clathrin-independent mechanism involving a novel cytoplasmic tripeptide motif of the receptor (Murphy *et al.*, 2008).

Much interest in LOX-1 has focused on its involvement in vascular disease, particularly the role of this receptor in the development of atherosclerosis. Many of the responses that are mediated by LOX-1 have been linked to pathological, proatherogenic, changes in the vascular endothelium, and the upregulation of LOX-1 in atherosclerotic lesions may result in a positive feedback loop that promotes disease development (Mehta *et al.*, 2006; Dunn *et al.*, 2008). In fact, increased release of soluble LOX-1 has been proposed to be a marker of acute coronary syndrome. Mouse models in which LOX-1 has been deleted, or overexpressed, also support a role for this receptor in the development of atherosclerosis (Inoue *et al.*, 2005; Mehta *et al.*, 2007; Hu *et al.*, 2008). Furthermore, although controversial, genetic linkage studies have implicated human polymorphisms of LOX-1 with susceptibility to cardiovascular disease (Mehta *et al.*, 2006; Dunn *et al.*, 2008; Knowles *et al.*, 2008). Finally there is also evidence that LOX-1 may be involved in thrombosis, myocardial ischemia reperfusion injury and hypertension and the receptor may be involved in generating inflammatory responses during microbial infection (Mehta *et al.*, 2006; Hu *et al.*, 2008).

## Concluding remarks

Despite considerable similarity in structure, the Dectin-1 cluster of receptors recognizes a diverse range of structurally unrelated ligands and mediates a wide variety of cellular functions. While our understanding is still in its infancy, the study of these receptors has revealed a number of new insights into the underlying mechanisms of immunity and homeostasis. One of the many remaining challenges is to fully elucidate the functions and ligands of the ‘orphan’ receptors within this cluster, a task that should be greatly aided by the generation and characterization of receptor deficient mice.

## Statement

Reuse of this article is permitted in accordance with the Creative Commons Deed, Attribution 2.5, which does not permit commercial exploitation.
